# To Mia or not to Mia: stepwise evolution of the mitochondrial intermembrane space disulfide relay

**DOI:** 10.1186/s12915-017-0468-1

**Published:** 2017-12-14

**Authors:** Chris Carrie, Jürgen Soll

**Affiliations:** 1 Department of Biology I, Botany, Ludwig-Maximilians-Universität München, Großhaderner Strasse 2-4, D-82152 Planegg-Martinsried, Germany; 2 Munich Center for Integrated Protein Science, CiPSM, Ludwig-Maximilians-Universität München, Feodor-Lynen-Strasse 25, D-81377 Munich, Germany

## Abstract

The disulfide relay system found in the intermembrane space (IMS) of mitochondria is an essential pathway for the import and oxidative folding of IMS proteins. Erv1, an essential member of this pathway, has been previously found to be ubiquitously present in mitochondria-containing eukaryotes. However, the other essential protein, Mia40, was found to be absent or not required in some organisms, raising questions about how the disulfide relay functions in these organisms. A recent study published in *BMC Biology* demonstrates for the first time that some Erv1 proteins can function in oxidative folding independently of a Mia40 protein, providing for the first time strong evidence that the IMS disulfide relay evolved in a stepwise manner.

See research article: 10.1186/s12915-017-0445-8

## Commentary

Eukaryotic cells have two separate compartments that contain disulfide relays in order to introduce disulfide bonds into proteins: the endoplasmic reticulum (ER) and the intermembrane space (IMS) of mitochondria [1]. In general, the principles underlying protein oxidation in both compartments are the same: at first the substrate protein is oxidized by oxidoreductases (mitochondrial import and assembly protein of 40 kDa (Mia40) in the IMS, protein disulfide isomerase (PDI) in the ER), which shuttle disulfide bonds that were initially generated by sulfhydryl oxidases (essential for respiration and viability 1 (Erv1) in the IMS and ER oxidoreductin 1 (Ero1) in the ER) [1]. The power of this process stems from the oxidizing effect of oxygen. Interestingly, recent work by Peleh et al. published in *BMC Biology* provides evidence that certain sulfhydryl oxidases from the IMS can promote the oxidative folding of substrates independently of any oxidoreductase [2]. This supports the idea that in the IMS of mitochondria the pathway evolved in a stepwise manner from a pathway utilizing only a sulfhydryl oxidase to one containing both a sulfhydryl oxidase and an oxidoreductase [3].

**Fig. 1:**
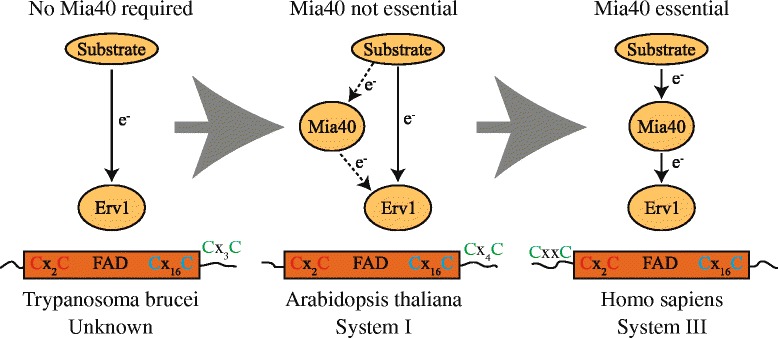
Stepwise evolution of the mitochondrial IMS disulfide relay. Displayed are the disulfide relay systems from three selected species, demonstrating the three potential phases of the stepwise evolution. Firstly, an Erv1-only relay as observed in *Trypanosoma brucei*. Secondly, a relay containing a Mia40 that is dispensable as observed in *Arabidopsis thaliana*. Thirdly, a relay with both an essential Mia40 and Erv1 as observed in *Homo sapiens*. Indicated for all organisms are the structural domain organization and the pairs of cysteine’s required for correct function of Erv1. Also indicated are the c-type cytochrome maturation pathways which function within each species

In the model systems of yeast and humans the mitochondrial IMS disulfide relay pathway contains two essential proteins: the oxidoreductase Mia40 and the FAD-containing sulfhydryl oxidase Erv1 [4]. After synthesis in the cytosol, mitochondrial IMS proteins are transported across the outer membrane in a reduced, unfolded state. In the IMS, substrates first interact with oxidized Mia40. Mia40 contains an essential redox active disulfide bond in a conserved cysteine-proline-cysteine motif that facilitates the stable folding of the substrate by the introduction of disulfide bonds, thus trapping the substrates within the IMS. Mia40 is then reoxidized by Erv1 [1, 4]. The active site of Erv1 is made up of two essential redox-active cysteine-x-x-cysteine pairs (where x is any amino acid) that shuttle the electrons from Mia40 to FAD [1, 4] (Fig. 1). To complete the disulfide relay Erv1 is oxidized by cytochrome c, which in turn passes the electrons via cytochrome c oxidase to oxygen. The latter reaction produces water rather than hydrogen peroxide, which would happen if oxygen is directly oxidized by Erv1, and it is also thought to increase the efficiency of Erv1 oxidation. While this is how the pathway functions in yeast and humans, significant differences were observed in wider phylogenetic groups. Examples of this are in the model plant *Arabidopsis thaliana*, where Mia40 was dispensable, and in earlier branching eukaryotes such as *Trypanosoma brucei*, *Encephalitozoon cuniculi*, and *Plasmodium falciparum* where no Mia40 proteins could be identified [3, 5–7]. Taken together with the knowledge that Erv1 homologs have been universally found in genomes of mitochondria-containing eukaryotes, this led to the hypothesis that either there were other unknown unidentified oxidoreductases in the IMS of some mitochondria or that some Erv1 proteins could react and oxidatively fold substrate proteins alone [3, 8].

The latter hypothesis was supported by the fact that, in plants, trypanosomes and *E. cuniculi* Erv1 contained a different primary structure. While all identified Erv1 proteins contained the conserved C-16x-C structural cysteine motif, the FAD binding domain and also the C-x-x-C motif adjacent to the FAD binding domain (Fig. 1), differences were observed with the final shuttle cysteine pair. In humans and yeast, where Mia40 is essential, the shuttle cysteine pair are located within the N-terminal part of the protein, while in eukaryotes either lacking a Mia40 or where Mia40 was not essential the shuttle cysteine pair was found to be located within the C-terminal part of Erv1 (Fig. 1). The exception to this is *Plasmodium*, which has an atypical arrangement of the cysteine pairs [6]. In the current study by Peleh et al. an *erv1* deletion strain was successfully complemented by the *Arabidopsis* Erv1 (AtErv1) protein, which is the first instance of such a distantly related protein being able to replace the yeast Erv1 [2]. While the complemented strain was not completely rescued it could be demonstrated that some IMS proteins were still detected, indicating that the AtErv1 could facilitate the import and folding of low levels of some essential IMS proteins. Interestingly it was also shown that AtErv1 could not oxidize the yeast Mia40 (ScMia40), indicating that AtErv1 alone was responsible for the oxidative folding of the IMS substrates within the complemented strain. To follow this up in vitro cytochrome c reduction studies demonstrated that AtErv1 could rapidly oxidize glutathione, leading to cytochrome c reduction, while in comparison ScErv1 was extremely inefficient in its interaction with glutathione. AtErv1 was also shown to be able to oxidize the well-characterized Mia40 substrate Cox19 in vitro. All this indicated that AtErv1 was able to oxidize IMS substrate proteins on its own by bypassing Mia40. To test this Peleh et al. demonstrated that, in both temperature-sensitive and redox-inactive *mia40* mutants, AtErv1 was able to partially suppress the growth defects and restore the reduced levels of several Mia40 substrate proteins [2], indicating that AtErv1 could directly interact and oxidatively fold IMS substrate proteins alone.

From this present study it can now be extrapolated that organisms lacking Mia40 proteins or that contain a non-essential Mia40 can utilize only Erv1 for oxidative folding within the IMS (Fig. 1). This strengthens the theory that the Mia40-Erv1 pathway evolved in a stepwise manner from an Erv1-only system, as previously hypothesized [3]. The stepwise evolution of the pathway may have evolved in the following three steps:

1. The ancestral or original IMS import pathway only contained an Erv1 protein, similar to the situation that is observed in present day eukaryotic groups such as *Leishmania* and *Trypansoma*, all of which lack genes for Mia40 homologs (Fig. 1). At this stage Erv1 would import and fold IMS proteins alone.
2. Later during evolution Mia40 was added, albeit firstly in a non-essential role, similar to the situation observed in present day plants (Fig. 1). At this stage Erv1 can still function alone, and the exact nature of the function of Mia40 is currently unclear. However, in yeast complementation experiments, AtErv1 still required Mia40 to perform its import function but not the oxidation function [2], hinting that Mia40 was first added as some kind of import chaperone before acquiring its essential oxidation function.
3. Finally during the evolution of fungi and animals the shuttle cysteines moved from within the C-terminus to the N-terminus of Erv1, and Erv1 lost its ability to oxidize substrate proteins alone, therefore requiring Mia40 to oxidize substrate proteins and become an essential part of the disulfide relay (Fig. 1).

It is important to understand that in no way does this model imply that fungi and mammals evolved from plants given the intermediate stage of the plant system, but more likely mammals and fungi developed their system from one similar to that which is still operating within plant mitochondria. The next question then is why did mammals and fungi add Mia40 to the disulfide relay system during evolution?

Here again Peleh et al. offer an interesting hypothesis. One of the major differences between the IMS of plants and that of mammals and fungi is that they use different systems for the biogenesis of c-type cytochromes [9]. Plant mitochondria still utilize the so-called system I or bacterial Ccm system. System I requires the activity of a variety of conserved proteins, many of which, in plants, are mitochondrially encoded, most likely due to their high hydrophobicity. In contrast mammals and fungi utilize the much simpler so-called system III for c-type cytochrome biogenesis, which requires only one protein: the cytochrome c heme lyase [9]. It appears that the addition of Mia40 to the disulfide relay system corresponds to the change in systems used for c-type cytochrome maturation. This possible connection between the disulfide relay system and c-type cytochrome biogenesis warrants further studies, including looking at interesting organisms, such as the green algae *Chlamydomonas reinhardtii*, which, while appearing to contain a plant type Erv1, actually utilizes the system III for c-type cytochrome biogenesis [9]. It is possible that Mia40 in *Chlamydomonas* is actually essential, in contrast to what was observed in *Arabidopsis*. But what does Mia40 bring to the disulfide relay that is not present within Erv1? Here a comparison with the ER is required. Within the ER it is presumed that the sulfhydryl oxidase Ero1 only oxidizes the single substrate PDI. Therefore, the specificity for substrates within the ER disulfide relay system is introduced at the oxioreductase level (PDI level). By adding Mia40 to the IMS pathway this may have increased substrate specificity of the disulfide relay as a whole, thereby allowing the development of a simpler maturation system (system III) for c-type cytochromes. Interestingly, the *Arabidopsis* CCMH protein, which is essential for c-type cytochrome maturation in plants, was found to be a potential substrate for AtErv1 [2]. The CCMH protein is only required in the bacterial system I maturation system so would not be a required substrate in system III mitochondria. This indicates that the significant evolutionary divergences in what was thought to be a conserved pathway for oxidative folding between different mitochondria may come down to the significant differences in IMS biochemistry.

The study by Peleh et al. demonstrates that some Erv1 proteins can interact and oxidatively fold substrate proteins in the absence of Mia40, answering questions of how plants could survive when Mia40 was knocked out and how eukaryotes which lack genes for Mia40 homologs could import and fold IMS proteins. This indicates that the IMS disulfide relay system most likely evolved in a stepwise manner. However, the study also brings up interesting questions like how does AtErv1 interact with substrate proteins? This is an intriguing question as much work has been performed on how Mia40 recognizes its substrate proteins. In fact, it is well recognized that the substrate specificity of Mia40 is achieved by the mitochondrial intermembrane space sorting signal/intermembrane space targeting signal (MISS/ITS), which are located on IMS proteins that require disulfide bonds. This recognition is mediated through a hydrophobic substrate binding cleft found within the structure of Mia40 [10]. How Erv1 mediates these same interactions is something for the future. Another interesting question raised by this work is how in the intermediate plant-like pathway Erv1 overcomes the observed effect of being a competitive inhibitor of Mia40. Studies looking at the interactions of plant Mia40 and Erv1 may help to understand how this incompatibility is overcome.
